# Design of Zeolite-Covalent Organic Frameworks for Methane Storage

**DOI:** 10.3390/ma13153322

**Published:** 2020-07-26

**Authors:** Ha Huu Do, Soo Young Kim, Quyet Van Le, Nguyen-Nguyen Pham-Tran

**Affiliations:** 1 Institute for Computational Science and Technology (ICST), Quang Trung Software City, Ho Chi Minh City 700000, Vietnam; hadohuu1311@gmail.com; 2 School of Chemical Engineering and Materials Science, Chung-Ang University, 84 Heukseok-ro, Dongjak-gu, Seoul 06974, Korea; 3 Department of Materials Science and Engineering, Korea University, 145 Anam-ro Seongbuk-gu, Seoul 02841, Korea; 4 Institute of Research and Development, Duy Tan University, Da Nang 550000, Vietnam; 5 Faculty of Chemistry, University of Science, VNU-HCM, Ho Chi Minh City 700000, Vietnam

**Keywords:** ZCOFs, methane storage, porous materials, simulation, design

## Abstract

A new type of zeolite-based covalent organic frameworks (ZCOFs) was designed under different topologies and linkers. In this study, the silicon atoms in zeolite structures were replaced by carbon atoms in thiophene, furan, and pyrrole linkers. Through the adoption of this strategy, 300 ZCOFs structures were constructed and simulated. Overall, the specific surface area of ZCOFs is in the range of 300–3500 m^2^/g, whereas the pore size is distributed from 3 to 27 Å. Furthermore, the pore volume exhibits a wide range between 0.01 and 1.5 cm^3^/g. Screening 300 ZCOFs with the criteria towards methane storage, 11 preliminary structures were selected. In addition, the Grand Canonical Monte Carlo technique was utilized to evaluate the CH_4_ adsorption ability of ZCOFs in a pressure ranging from 1 to 85 bar at a temperature of 298 K. The result reveals that two ZCOF structures: JST-S 183 *v/v* (65–5.8 bar) and NPT-S 177 *v/v* (35–1 bar) are considered as potential adsorbents for methane storage. Furthermore, the thermodynamic stability of representative structures is also checked base on quantum mechanical calculations.

## 1. Introduction

Methane is known as the fundamental ingredient of natural gas which is found with oil fields in Earth’s crust. Currently, methane is also exploited from methane hydrate, recognized as a promising resource to provide energy in the future. However, efficiency and safety are two critical criteria for methane storage that need to be addressed in practical applications. Therefore, a large number of studies have focused on methane storage with porous materials such as covalent organic frameworks (COFs), zeolitic imidazole frameworks, hydrogen-bonded organic frameworks, and metal-organic frameworks (MOFs) [1,2,3,4,5,6]. In 2006, ZIFs were firstly reported with various topologies such as sod, rho, and mer [7]. This study opened a new window for the synthesis of porous material as well as their applications. In addition, zeolite-like MOFs (ZMOFs) were also simulated and prepared with different zeolite frameworks [8,9]. For example, two anionic ZMOFs, including rho-ZMOF and sod-ZMOF were fabricated successfully by Liu et al. [8]. These materials have a high surface area which can provide exceptional gas adsorption such as H_2_, CH_4_, and CO_2_. Therefore, ZCOFs are evaluated as potential porous materials for gas storage.

Over the past decade, COFs have become interesting materials in the porous material group due to their outstanding specific surface area. Similar to MOF, COFs have crystal structures with controllable pore sizes [10]. However, COFs have a definite advantage that they only contain non-metal elements such as C, Si, B, O, and H. These elements were linked together by a vast number of covalent bonds to form COFs structures. In 2005, the first COF materials were synthesized by Yaghi et al., using the solvothermal method [10]. Later, many researchers tried to expand the ability to synthesize COFs in different ways [11]. Similar to MOFs, COFs have been scrutinized in a wide range of utilizations such as gas storage [12,13,14,15,16], catalysis [17,18], and sensors [19,20] due to their feature properties such as high thermal durability, large surface area, and low density [15]. For example, COF-108 displayed a very low density of 0.17 g·cm^−3^ which is lower than any reported materials [21,22,23]. COF-105 gave a high surface area of over 6000 m^2^·g^−1^ [21]. The US Department of Energy (DOE) proposed a standard of the CH_4_ adsorption ability for porous materials to be 180 V(STP)/V, (where STP is the standard temperature and pressure) [24]. On the theoretical study side, Goddard et al. successfully designed two materials with appropriate functional groups to enhance the methane storage properties of some COF materials [25]. That was, COF-103-Eth-trans and COF-102-Ant with methane adsorption capacity exceeding the target set by the DOE (Figure S1). Another group, Jing Hao Hu et al., modified COF-102 with a double halogen substitution [26]. Their simulation result shows that the methane adsorption capacity of COF-102-1,4-2I is 181 V (STP)/V. In addition, a few porous materials have been fabricated experimentally which surpassed the DOE standard, such as Ni-MOF-74 [27] (190 v(STP)/v) and PCN-14 [28] (220 v(STP)/v).

Recently, a new DOE target has been provided for methane storage to be 315 cm^3^/cm^3^ (at (35–1 bar) or (65–5.8 bar)) for the single crystal material [29,30]. To date, there was not any experimental COFs, which can overcome the DOE target. Therefore, several efforts related to the porous materials were conducted to find outstanding candidates for methane storage. For instance, Zhao et al. used the various functional groups involving –CF_3_, –CH_3_, -CN, -OCH_3_, -CN, -Cl, Br, I, and NH_2_ to improve the methane adsorption ability of three-dimensional COFs [31]. The result indicated that COF-102-I exhibited the highest CH_4_ uptake among the modified materials. Martin et al. generated a large number of porous polymer networks (~18,000 structures) for CH_4_ adsorption. However, only three structures achieved the CH_4_ adsorption of 180 cm^3^/cm^3^ [32]. This result implied that finding excellent materials for methane storage is a great challenge for scientists. Herein, under the support of computer tools, we proposed a strategy design of COF materials with different zeolite frameworks to find potential COF materials that are capable of methane storage reaching the DOE target. We also demonstrate the survival of selected COFs from rational design, which provides useful information for experimental studies.

## 2. Design Strategy and Methodologies

**Figure 1:**
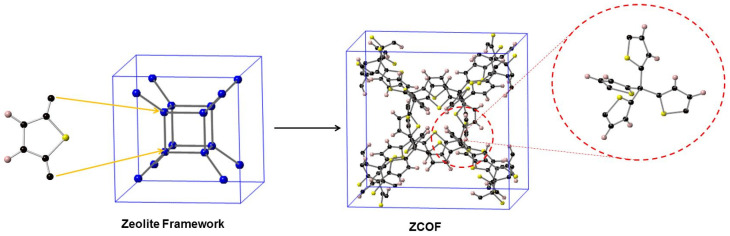
Graphical illustration of the design strategy for zeolite-based covalent organic frameworks (ZCOFs) materials. The spheres in black, pink, and yellow denote C, H, and S (or O, N-H) atoms, respectively.

Figure 1 illustrates the designed strategy of the covalent organic framework from the zeolite frameworks. Replacing the silicon atom in 100 selected zeolite framework types by carbon from thiophene (Figure S2), furan, and pyrrole linkers, 300 ZCOF structures were constructed. Since the topology of ZCOFs is inherited by the zeolite framework types, we named the newly designed materials, such as YYY-S, YYY-O, and YYY-N. Therein, YYY stands for the framework type code of the zeolite, -S, -O, -N stand for thiophene, furan, and pyrrole, respectively.

The ZCOF structures are built and optimized through the universal force field via forcite tools [33]. The pore diameter (Dpore), accessible surface area (Sacc), and pore volume (Vpore) are calculated by the ZeO++ code. A spherical model with a radius of 1.8405 Å was used to simulate the N_2_ molecule [34,35].

All grand canonical Monte Carlo (GCMC) computations were conducted by utilizing the MUSIC code [36]. The potential energy between the COF-CH_4_ and CH_4_-CH_4_ was obtained from van der Waals interactions since CH_4_ is the poor polar molecule. The CH_4_ molecule is simulated as a sphere with a kinetic diameter of 3.8 Å [37]. Lennard-Jones potential was used with the parameters acquired from the transferable force fields (TraPPE) [38] for CH_4_ and Universal Force field for the atoms in the ZCOFs [33]. The distances are more considerable than 12.8 Å, not considered in this model. The parameters for interactions between the atoms of ZCOFs and CH_4_ molecules were estimated through the Lorentz-Berthelot rule [39]. In the simulation, a supercell 2 × 2 × 2 of COF was kept rigid, CH_4_ was considered a ‘spherical molecule’. For each pressure point, 15 × 106 Monte Carlo trial moves were performed. This technique has been successfully applied for adsorption studies, reported in the previous works [40]. The variables of force field for ZCOFs and CH_4_ are provided in Table 1. The detailed parameters and structures of ZCOF were shown in Table S1 in the supplementary information.

Implementing the density functional theory (DFT) for periodic systems in CRYSTAL17 was used for the study of the structural stability of new ZCOFs [41,42]. Specifically, the calculations were executed with the exchange-correlation functional of Perdew-Burke-Ernzerhof (PBE), and a basic set of 6-31G functions for atoms in the ZCOF structures. The values of 0.00030 and 0.00045 are indications of the convergence criteria of force for a root-mean-square and maximum component of the gradient, respectively, whereas, the condition was set to 10^−7^ Hatrees during the geometry optimization for self-consistent total energy calculations (NPT-S, JST-S code).

## 3. Results and Discussion

### 3.1. Screening ZCOFs for Methane Storage

**Figure 2:**
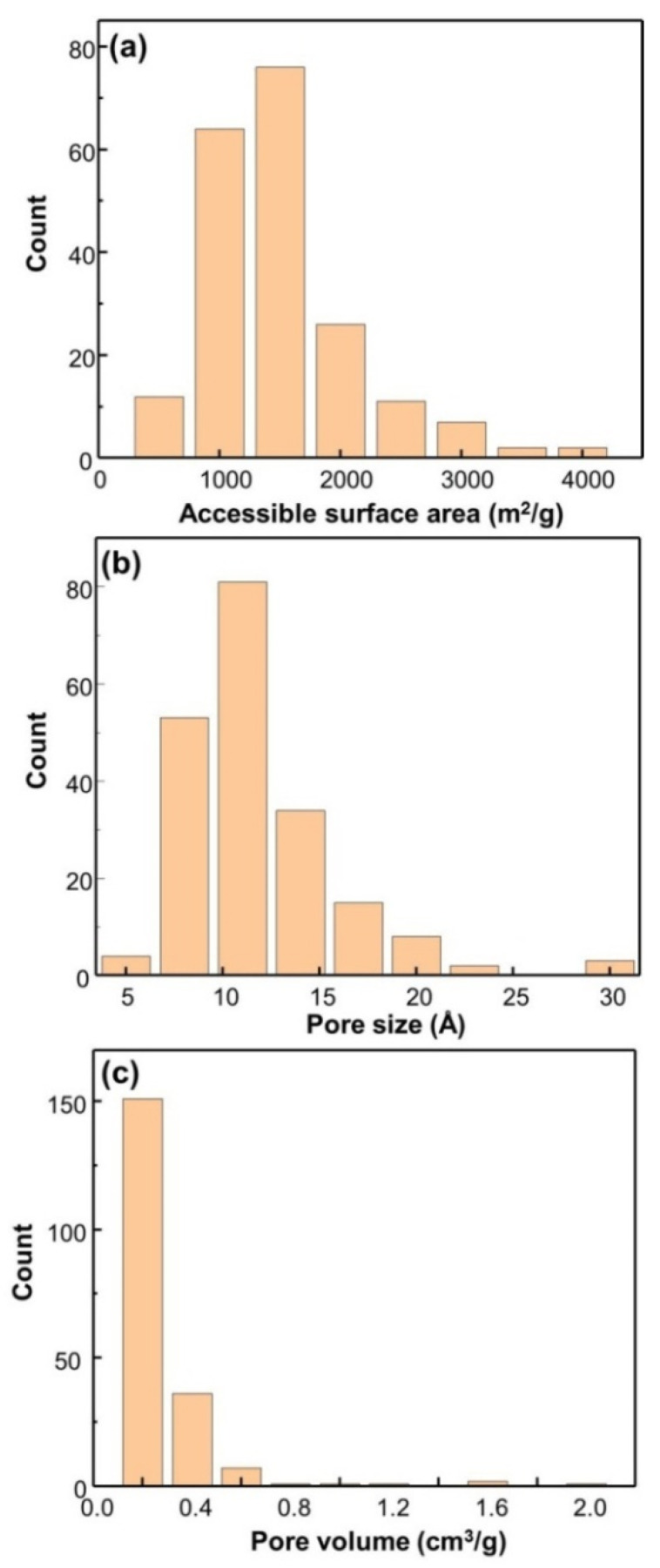
Statistical analysis of all 300 ZCOFs constructed: (**a**) Accessible surface area; (**b**) pore size; (**c**) pore volume.

Figure 2 provides information about Dpore, Sacc, and Vpore of 300 ZCOF structures. The pore sizes of these ZCOFs cover a range from 3 to 27 Å, and most ZCOFs have a pore size of about 8–14 Å, whereas the distribution of the surface area revealed that most ZCOFs exhibited the high surface area between 1000 and 2000 m^2^/g and extended to nearly 3500 m^2^/g. This is well because the Sacc of zeolite is often lower than 900 m^2^/g. Notably, RWY-N displayed the largest surface area of 3437 m^2^/g among 300 ZCOF materials. However, the pore volume of ZCOF is not much higher than that of zeolite and most are less than 1 cm^3^/g.

Using the screening parameters for methane storage materials in the work of Martin et al. [32], such as Dpore > 10 Å, Sacc > 2000 m^2^/g, and Vpore > 0.4 cm^3^/g, a total of 11 ZCOF candidates were selected through 300 designed ZCOFs. The parameters of 11 ZCOF structures are provided in Table 2.

### 3.2. Adsorption of CH_4_

**Figure 3:**
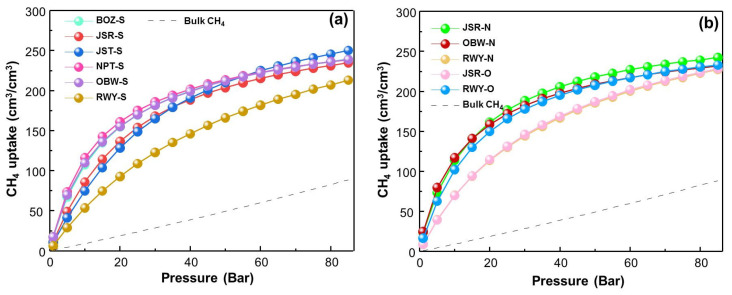
Isotherms of total CH_4_ adsorption in a pressure ranging from 1 to 85 bar at a temperature of 298 K of 11 ZCOFs. (**a**) XXX-S; (**b**) XXX-O and XXX-N.

**Figure 4:**
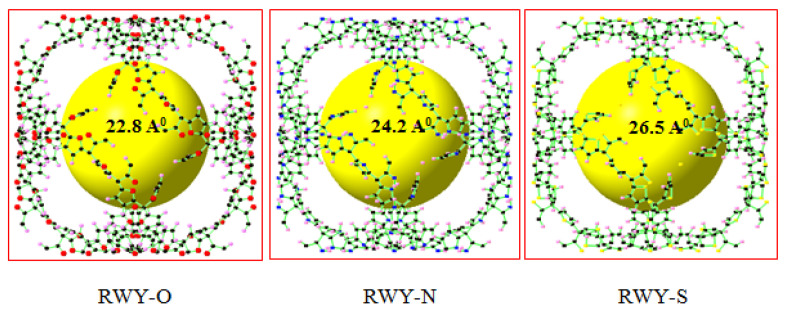
ZCOF crystal structures of RWY-O, RWY-N, and RWY-S. The spheres in black, pink, red, blue, and orange denote C, H, O, N, and S atoms, respectively. The yellow balls represent the pore size.

The CH_4_ adsorption capacity at 298 K for the 11 selected ZCOFs was illustrated in Figure 3. We realize that the adsorption isotherm of the selected ZCOFs is quite close together. Methane adsorption of most of the ZCOFs rapid growth in the range of 0–20 bar then increases slowly and reaches equilibrium at a pressure of about 60 bar. However, three ZCOF crystal structures: RWY-O, RWY-N, and RWY-S with an increasing pore diameter (as shown in Figure 4, Figure S3), have shown lower methane uptake compared to the other ZCOFs. This can be explained via their pore size, the ZCOF with a large pore size increases the distance of methane and active sites, leading to the weak interactions. Thus, the too-large pore diameter was not favorable in the methane storage application.

In 11 selected ZCOF structures, JSR-N has the highest adsorption capacity at 35 bar with 197 and 231 (*v/v*) at 65 bar and followed by BOZ-S, OBW-S, JSR-O, and OBW-N. In addition, the bulk density of methane was also illustrated by a black dotted line for comparison purposes. It is clearly shown that ZCOFs are effective adsorbents for methane storage applications.

### 3.3. Methane Delivery Capacity

The methane delivery capacity of the ZCOF selected structures is calculated as follows [32]:

DC (35–1) = the CH_4_ adsorption at 35 bar − the CH_4_ adsorption at 1 bar (1)

DC (65–5.8) = the CH_4_ adsorption at 65 bar − the CH_4_ adsorption at 5.8 bar (2)

**Figure 5:**
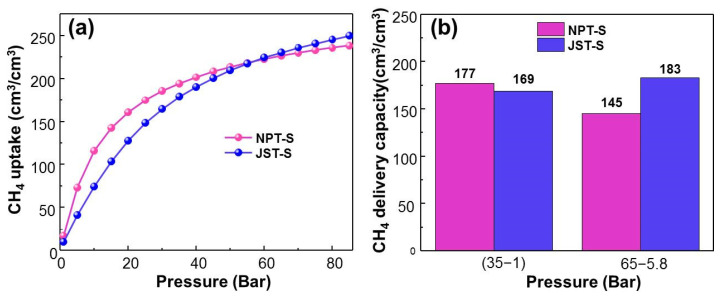
(**a**) Isotherms of total volumetric CH_4_ uptake at 298 K from 0 to 85 bar, and (**b**) CH_4_ delivery capacity of NPT-S and JST-S.

**Figure 6:**
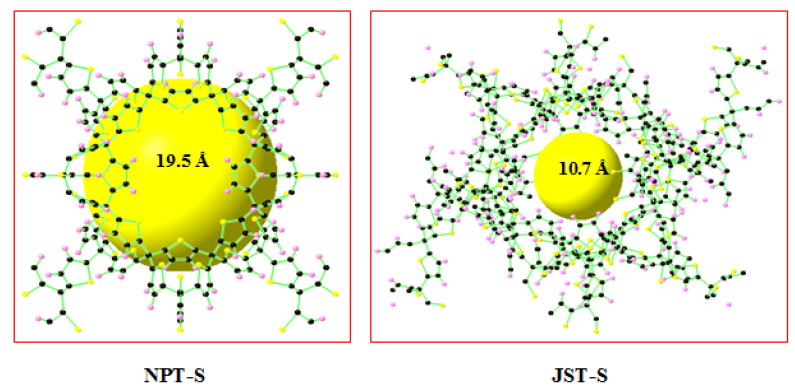
Two ZCOF structures: NPT-S and JST-S. The spheres in black, pink, and orange denote C, H, and S atoms, respectively. The yellow balls represent the pore size.

Table 2 presents the delivery capacity of 11 ZCOF structures, which exhibited excellent performances for methane storage. In total, NPS-S and JST-S give the best methane uptake, as shown in Figure 5a. In particular, Figure 5b indicated that NPT-S gave the largest DC (35–1) of 177 cm^3^_STP_ (CH_4_)/cm^3^, while the largest DC (65–5.8) reached was 183 cm^3^_STP_ (CH_4_)/cm^3^, for the JST-S structure. The results are comparable with the previous studies and DOE 2000 target for methane storage (180 cm^3^_STP_/cm^3^), as displayed in Table 3 [43]. This finding was attributed to the appropriate porous parameters for methane storage, as reported in Martin’s study [32]. Notably, JST-S has a pore size of 10.7 Å, whereas NPT-S gave a pore diameter of 19.5 Å, as illustrated in Figure 6.

### 3.4. CH_4_ Adsorption Sites

**Figure 7:**
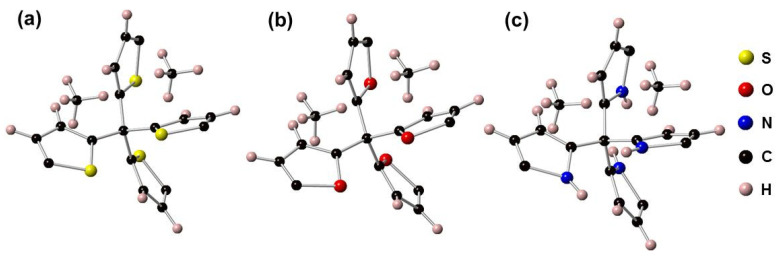
The adsorption of CH_4_ on the surface of thiophene (**a**), furane (**b**), and pyrrole rings (**c**) for YYY-S, YYY-O, and YYY-N, respectively.

To date, only several studies provide the mechanism for CH_4_ adsorption in porous materials. For instance, Mendoza-Cortes et al. indicated that the various 3D-COFs, including COF-105, COF-103, and COF-102, contains the adsorption centers on the surface of the benzene ring [5]. In this work, we propose that the CH_4_ adsorption sites can be on the face of the thiophene, furane, and pyrrole rings, as illustrated in Figure 7.

### 3.5. The Formation Energy of JST-S and NPT-S

The heat of formation is a powerful means to predict the thermodynamic stability of any structures. The enthalpy of formation with a negative value indicates that the considered compound is stable in terms of thermodynamic. In this research, the reaction enthalpy for JST-S and NPT-S generation was determined from the change in the total enthalpy between the products and reactants. They were calculated from reaction (3) and (4):

18 Cgraphite + 4 H_2_ + 1/2 S_8_ → C_18_H_8_S_4_ (JST-S) (3)

54 Cgraphite + 12 H_2_ + 3/2 S_8_ → C_54_H_24_S_12_ (NPT-S) (4)

The reactants selected to be the best stable state in nature, include graphite, hydrogen gas, and sulfur (rhombic). Therefore, their standard enthalpy of formations is zero. The negative quantities exhibited in Table 4, implied that JST-S and NPT-S could be synthesized in the experiment.

## 4. Conclusions

In summary, we have shown the design strategy of a new covalent organic framework by using carbon to replace silicon in zeolite through thiophene, furan, and pyrrole linkers, named so we can obtain 300 ZCOF structures with 100 topologies of zeolite. The typical porous parameters, including the accessible surface area, pore size, and pore volume were analyzed to evaluate the quality of ZCOFs material through the Zeo++ software. The results reveal that the surface area of ZCOFs is larger than that of zeolites, which is favorable for methane storage. Notably, RWY-N displayed the largest accessible surface area of 3437 m^2^/g, proposed for the promising material in gas storage applications. Among 300 ZCOFs materials, eleven optimal structures involving BOZ-S, JSR-N, JSR-O, JSR-S, JST-S, NPT-S, OBW-N, OBW-S, RWY-N, RWY-O, and RWY-S exhibited good methane adsorption ability. In particular, NPT-S displayed the largest DC (35–1) of 177 cm^3^_STP_ (CH_4_)/cm^3^, whereas JST-S exhibited the best DC (65–5.8) of 183 cm^3^_STP_ (CH_4_)/cm^3^. This finding can be comparable to the old DOE standard. In addition, the calculation of enthalpy of NPT-S and JST-S was also implemented by the DFT method, showing negative values. This result implied that these structures could be prepared in the experimental study.

## Figures and Tables

**Table 1:** The parameters of force field for ZCOF and CH_4_.

Molecule	Atom	ε/kb (K)	σ (Å)	ref
**CH_4_**	-	148.0	3.73	[38]
**ZCOFs**	C	52.8	3.43	[33]
	H	22.1	2.57	-
	S	137.9	3.59	-
	O	30.2	3.12	-
	N	34.7	3.26	-

**Table 2:** The porous properties, total methane uptake, and delivery capacity of the 11 selected ZCOF structures.

ZCOFs	Sacc (m^2^/g)	Dpore (Å)	Vpore (cm^3^/g)	Total Uptake at 35 bar (cm^3^/cm^3^)	Delivery Capacity 35–1 bar (cm^3^/cm^3^)	Total Uptake at 65 bar (cm^3^/cm^3^)	Delivery Capacity 65–5.8 bar
BOZ-S	2579	16.1	0.516	191	174	227	152
JSR-N	2865	12.8	0.543	197	174	231	150
JSR-O	2702	12.4	0.489	187	170	221	151
JSR-S	2480	13.6	0.612	179	168	220	164
JST-S	2615	10.7	0.433	179	169	230	183
NPT-S	2548	19.5	0.502	194	177	227	145
OBW-N	2920	13.8	0.451	191	166	221	134
OBW-S	2734	15.7	0.557	191	174	226	148
RWY-N	3437	24.2	1.212	156	147	206	161
RWY-O	3273	22.8	1.055	158	149	208	163
RWY-S	3209	26.5	1.419	135	129	189	156
Bulk CH_4_	-	-	-	34	33	66	61

**Table 3:** Comparison of the methane uptake of NPT-S and the other COFs at 35 bar and 298 K.

ZCOFs	Sacc (m^2^/g)	Dpore (Å)	Vpore (cm^3^/g)	CH_4_ Uptake (cm^3^/cm^3^)	CH_4_ Delivery (cm^3^/cm^3^)	Ref
COF-102-Ant	2720	-	0.75	215	180	[25]
COF-103-Eth-trans	4920	-	1.36	206	192	[25]
COF-102-1,4-2I	-	-	-	-	181	[26]
COF-102-I	-	-	-	176	169	[31]
COF-102-Cl	-	-	-	169	165	[31]
COF-1	750	9	0.30	55	-	[44]
COF-5	1670	27	1.07	73	-	[44]
COF-6	750	9	0.32	101	-	[44]
COF-8	1350	16	0.69	85	-	[44]
COF-10	1760	32	1.44	53	-	[44]
COF-102	3620	12	1.55	113	-	[44]
COF-103	3530	12	1.54	105	-	[44]
**NPT-S**	**2548**	**19.5**	**0.502**	**194**	**177**	**This work**

**Table 4:** Optimized crystal structure lattice parameters.

ZCOF	Symmetry	Atom/Cell	Lattice Parameter (Å)	∆H (kJ/mol)
JST-S	Pa-3	720	27.3204	−34,823 × 10^3^
NPT-S	Pm-3	540	25.0125	−8705 × 10^3^

## Supplementary Materials

The following are available online at https://www.mdpi.com/1996-1944/13/15/3322/s1, Figure S1: Design strategy of ZCOFs with thiophene linker. The spheres in black, pink, yellow denote C, H, and S atoms, respectively, Figure S2: Isotherms of total volumetric CH_4_ uptake at 298 K from 0 to 85 bar of (a) RWY-X, and (b) JRN-X (X = N, O, S), Figure S3: (a) ctn and bor topologies, (b) the building blocks for designing new COFs, (c) the reactions from various reactants to created new COFs, Table S1: The parameter of 300 Z-COF structures, Computer Code: NPT-S, JST-S.
