# Supplementary material for: Design of Zeolite-Covalent Organic Frameworks for Methane Storage
Source: Materials (Basel). 2020 Jul 26;13(15):3322. doi: 10.3390/ma13153322 (PMC7435647; doi:10.3390/ma13153322)
Supplement: Supplementary file 1 [file materials-13-03322-s001.pdf]

materials

Supplementary Materials

Design of Zeolite-Covalent Organic Frameworks for Methane Storage

Ha Huu Do [1,2], Soo Young Kim [3,*], Quyet Van Le [4,*] and Nguyen-Nguyen Pham-Tran [1,5,*]

1 Institute for Computational Science and Technology (ICST), Quang Trung Software City, Ho Chi Minh City 700000, Vietnam; hadohuu1311@gmail.com
2 School of Chemical Engineering and Materials Science, Chung-Ang University, 84 Heukseok-ro, Dongjak-gu, Seoul 06974, Republic of Korea
3 Department of Materials Science and Engineering, Korea University, 145 Anam-ro Seongbuk-gu, Seoul 02841, Republic of Korea
4 Institute of Research and Development, Duy Tan University, Da Nang 550000, Vietnam
5 Faculty of Chemistry, University of Science, VNU-HCM, Ho Chi Minh City 700000, Vietnam
* Correspondence: sooyoungkim@korea.ac.kr (S.Y.K); levanquyet@dtu.edu.vn (Q.V.L.); ptnnguyen@hcmus.edu.vn (N-N P-T.)

Received: 26 June 2020; Accepted: 24 July 2020; Published: date

Table S1. The parameter of 300 Z-COF structures.

No.	Structure	Space group	ASA (m^2/g)	D pore (A°)	V-pore(cm^3/g)
1	ABW-S	Pmna	1067.4	6.2	0.05
2	AEI-S	Cmcm	1908.8	12.6	0.26
3	AEL-S	Pmna	713.5	9.1	0.08
4	AET-S	$C222_1$	745.5	14.8	0.20
5	AFO-S	$C222_1$	666.9	8.6	0.10
6	AFR-S	Pmmn	1560.5	13.6	0.27
7	AHT-S	$C222_1$	1132.1	6.9	0.09
8	APC-S	Ia-3d	1785.7	8.7	0.14
9	APD-S	Pmna	930.6	6.5	0.04
10	ASV-S	Pbca	1211.3	10.5	0.14
11	ATN-S	I4m2	1057.1	9.5	0.10
12	ATS-S	Pbcn	1120.3	12.1	0.21
13	ATT-S	Pm2a	1369.1	9.5	0.11
14	ATV-S	C2mb	609.0	6.1	0.04
15	AWO-S	Cmca	1322.0	8.5	0.12
16	AWW-S	P4/nmm	1292.3	12.0	0.20
17	BCT-S	I-4m2	520.4	6.0	0.02
18	BEC-S	P42/m	1500.1	11.7	0.31
19	BIK-S	$C222_1$	900.1	7.0	0.07
20	BOF-S	Pnma	1081.7	8.5	0.12
21	BOG-S	Imma	1352.8	14.3	0.28
22	BOZ-S	Pbcn	2579.2	16.1	0.52
23	CAS-S	$C222_1$	845.4	6.8	0.06

24	CDO-S	$C222_1$	1152.0	9.0	0.11
25	CFI-S	Pmna	886.9	12.0	0.18
26	CGS-S	Pnma	1520.8	10.0	0.22
27	DFT-S	P42/m	1316.4	7.4	0.10
28	DON-S	Pbcn	823.9	14.2	0.18
29	EDI-S	P-4	2049.0	8.2	0.16
30	EEI-S	F222	921.4	11.1	0.12
31	ESV-S	Pnma	1367.2	10.3	0.16
32	EUO-S	C2mb	1001.9	11.3	0.15
33	EZT-S	Pnma	1311.1	10.7	0.20
34	FER-S	I222	1228.4	9.7	0.13
35	GIS-S	Ia-3d	2026.9	8.8	0.17
36	GON-S	C222	736.7	9.6	0.08
37	GOO-S	$C222_1$	1519.6	8.2	0.13
38	IFO-S	Pnnm	1126.9	13.4	0.24
39	IHW-S	Pbca	953.9	10.4	0.12
40	IMF-S	Pbcn	1072.6	11.1	0.15
41	IRN-S	I222	1462.3	14.5	0.24
42	ITE-S	Cmcm	1460.4	13.9	0.24
43	ITH-S	Pnc2	1183.3	11.0	0.16
44	ITR-S	Pbcn	1164.0	10.7	0.18
45	IWR-S	C222	1433.6	13.2	0.30
46	IWV-S	F222	1345.0	14.7	0.33
47	IWW-S	Pba2	1373.9	11.9	0.20
48	JBW-S	$P2_122$	893.7	7.0	0.06
49	JOZ-S	$Pbc2_1$	2168.8	7.8	0.16
50	JRY-S	$I2_12_12_1$	1211.6	7.7	0.11
51	JSW-S	Pbca	1225.2	8.7	0.12
52	LOV-S	P42/mmc	1993.6	7.9	0.15
53	MER-S	I4/mmm	1845.1	11.0	0.20
54	MFI-S	Pnma	1107.0	10.9	0.15
55	MFS-S	Pnn2	1074.0	10.2	0.12
56	MOR-S	Pbcn	1237.9	10.3	0.15
57	MRE-S	Pmna	661.9	9.8	0.07
58	MTT-S	$P2_12_12$	857.2	9.4	0.08
59	MVY-S	Pnn2	962.3	6.7	0.07
60	NAB-S	I-4	2203.7	6.8	0.16
61	NES-S	F222	1168.7	11.9	0.18
62	NON-S	F222	881.8	9.7	0.10
63	OBW-S	Immm	2734.5	15.7	0.56
64	OWE-S	Pm2a	1352.2	8.7	0.09
65	PHI-S	Pbcn	1184.2	7.9	0.05
66	PON-S	$Pca2_1$	1329.8	8.1	0.07

67	POS-S	Pnnm	1126.0	11.4	0.96
68	PUN-S	Pbcn	2214.6	10.1	0.23
69	SAF-S	I222	895.4	13.1	0.10
70	SAO-S	I-4m2	1706.8	14.1	0.37
71	SAS-S	I4/m	1457.9	13.8	0.18
72	SAV-S	P4/nmm	1721.1	14.1	0.33
73	SBE-S	Immm	1790.4	21.5	0.41
74	SFG-S	$P2_122$	921.7	10.0	0.13
75	SFH-S	$C222_1$	1005.9	13.9	0.17
76	SIV-S	$C222_1$	1797.0	7.8	0.17
77	SZR-S	C222	1154.7	11.0	0.15
78	TER-S	$C222_1$	1230.2	10.2	0.15
79	THO-S	$P2_122$	1873.2	8.5	0.13
80	TON-S	$C222_1$	842.8	8.3	0.10
81	UEI-S	Fmm2	1362.1	8.3	0.07
82	UFI-S	Immm	1660.0	17.4	0.21
83	UOS-S	Pm2a	1363.0	8.9	0.22
84	VET-S	P-4	651.0	10.8	0.09
85	ZON-S	Pbcm	1371.1	9.7	0.16
86	ANA-S	Ia-3d	1242.2	6.7	0.06
87	AST-S	Pccn	1088.3	10.6	0.12
88	FAU-S	Fd-3m	1533.9	18.6	0.44
89	GIE-S	Ia-3d	1509.2	10.5	0.21
90	JSR-S	Pa-3	2480.1	13.6	0.61
91	JST-S	Pa-3	2614.8	10.7	0.43
92	KFI-S	Im-3m	1636.8	17.8	0.27
93	LTA-S	P432	1481.0	19.0	0.37
94	NPT-S	Pm-3	2548.1	19.5	0.50
95	MEP-S	P222	0.0	8.2	0.00
96	PCB-S	Pnnm	1053.4	6.8	0.02
97	RHO-S	Im-3m	1602.6	17.4	0.27
98	RWY-S	I-43m	3209.1	26.5	1.42
99	SOD-S	I432	2177.6	10.5	0.17
100	LTJ-S	$P4_12_12$	0.0	4.8	0.00
101	ABW-O	Pmna	0.0	4.9	0.00
102	AEI-O	Cmcm	1534.0	9.8	0.15
103	AEL-O	Pmna	575.5	7.8	0.05
104	AET-O	$C222_1$	702.1	11.3	0.13
105	AFO-O	$C222_1$	533.6	6.5	0.03
106	AFR-O	Pmmn	1199.2	12.5	0.17
107	AHT-O	$C222_1$	569.1	5.7	0.02
108	APC-O	Ia-3d	1382.6	7.3	0.08
109	APD-O	Pmna	555.0	5.3	0.02

110	ASV-O	Pbca	1103.5	9.3	0.10
111	ATN-O	I-4m2	572.3	7.8	0.03
112	ATS-O	Pbcn	1054.8	9.8	0.14
113	ATT-O	Pm2a	1246.5	7.3	0.07
114	ATV-O	C2mb	336.1	5.4	0.01
115	AWO-O	Cmca	909.8	6.9	0.05
116	AWW-O	P4/nmm	992.0	10.5	0.10
117	BCT-O	I-4m2	0.0	5.2	0.00
118	BEC-O	P42/m	1400.7	9.7	0.22
119	BIK-O	$C222_1$	806.4	6.0	0.04
120	BOF-O	Pnma	630.1	5.6	0.02
121	BOG-O	Imma	1199.8	12.7	0.19
122	BOZ-O	Pbcn	2475.5	14.1	0.32
123	CAS-O	$C222_1$	0.0	4.8	0.00
124	CDO-O	$C222_1$	925.6	7.2	0.05
125	CFI-O	Pmna	934.0	10.8	0.12
126	CGS-O	Pnma	1028.4	7.8	0.08
127	DFT-O	P42/m	963.1	6.5	0.05
128	DON-O	Pbcn	713.0	10.9	0.14
129	EDI-O	P-4	1336.6	7.2	0.07
130	EEI-O	F222	0.0	9.3	0.00
131	ESV-O	Pnma	889.9	8.8	0.08
132	EUO-O	C2mb	924.4	9.5	0.09
133	EZT-O	Pnma	922.5	9.3	0.11
134	FER-O	I222	1094.0	8.3	0.08
135	GIS-O	Ia-3d	0.0	6.5	0.00
136	GON-O	C222	642.0	8.3	0.05
137	GOO-O	$C222_1$	1029.2	6.8	0.06
138	IFO-O	Pnnm	878.7	11.5	0.18
139	IHW-O	Pbca	710.7	8.3	0.06
140	IMF-O	Pbcn	930.4	9.3	0.09
141	IRN-O	I222	1012.2	12.3	0.14
142	ITE-O	Cmcm	1311.5	11.5	0.16
143	ITH-O	Pnc2	922.5	8.8	0.08
144	ITR-O	Pbcn	1029.2	8.3	0.09
145	IWR-O	C222	1332.1	11.3	0.19
146	IWV-O	F222	1244.0	13.0	0.24
147	IWW-O	Pba2	1172.5	10.3	0.13
148	JBW-O	$P2_122$	551.4	5.5	0.02
149	JOZ-O	$Pbc2_1$	799.3	5.7	0.02
150	JRY-O	$I2_12_12_1$	*765.9*	5.6	0.03
151	JSW-O	Pbca	0.0	7.3	0.00
152	LOV-O	P42/mmc	1635.5	7.6	0.08

153	MER-O	I4/mmm	1360.1	10.1	0.10
154	MFI-O	Pnma	955.6	8.9	0.08
155	MFS-O	Pnn2	827.5	8.7	0.06
156	MOR-O	Pbcn	905.1	9.1	0.09
157	MRE-O	Pmna	530.8	8.1	0.05
158	MTT-O	$P2_12_12$	504.4	7.1	0.04
159	MVY-O	Pnn2	765.0	6.4	0.04
160	NAB-O	I-4	1829.4	5.8	0.07
161	NES-O	F222	1010.7	10.0	0.13
162	NON-O	F222	0.0	9.0	0.00
163	OBW-O	Immm	2688.9	13.3	0.37
164	OWE-O	Pm2a	1071.3	7.5	0.07
165	PHI-O	Pbcn	533.8	5.0	0.01
166	PON-O	$Pca2_1$	669.1	6.1	0.03
167	POS-O	Pnnm	906.8	10.1	0.09
168	PUN-O	Pbcn	2155.8	8.1	0.20
169	SAF-O	I222	719.6	12.1	0.11
170	SAO-O	I-4m2	1697.9	12.7	0.32
171	SAS-O	I4/m	1170.4	12.3	0.15
172	SAV-O	P4/nmm	1543.7	12.6	0.17
173	SBE-O	Immm	1610.7	19.4	0.34
74	SFG-O	$P2_122$	845.9	8.1	0.07
175	SFH-O	$C222_1$	918.3	12.2	0.16
176	SIV-O	$C222_1$	689.5	6.2	0.02
177	SZR-O	C222	1128.7	8.7	0.07
178	TER-O	$C222_1$	1075.0	8.6	0.08
179	THO-O	P2122	1333.9	6.2	0.07
180	TON-O	$C222_1$	545.5	6.4	0.03
181	UEI-O	Fmm2	0.0	6.9	0.00
182	UFI-O	Immm	1506.8	15.0	0.22
183	UOS-O	Pm2a	854.5	7.2	0.05
184	VET-O	P-4	514.6	9.3	0.06
185	ZON-O	Pbcm	1017.9	8.4	0.08
186	ANA-O	Ia-3d	0.0	5.0	0.00
187	AST-O	Pccn	1491.2	10.6	0.16
188	FAU-O	Fd-3m	1612.3	16.8	0.40
189	GIE-O	Ia-3d	1363.9	8.5	0.12
190	JSR-O	Pa-3	2702.3	12.4	0.49
191	JST-O	Pa-3	2842.1	8.7	0.22
192	KFI-O	Im-3m	1406.7	16.3	0.21
193	LTA-O	P432	1196.0	15.5	0.21
194	NPT-O	Pm-3	2479.7	17.1	0.36
195	MEP-O	P222	0.0	6.8	0.00

196	PCB-O	Pnnm	655.4	6.5	0.03
197	RHO-O	Im-3m	1385.5	14.4	0.17
198	RWY-O	I-43m	3272.6	22.8	1.05
199	SOD-O	I432	2326.3	10.9	0.23
200	LTJ-O	$P4_12_12$	0.0	3.6	0.0
201	ABW-N	Pmna	0.0	5.7	0.00
202	AEI-N	Cmcm	1758.1	10.6	0.19
203	AEL-N	Pmna	594.0	8.2	0.05
204	AET-N	$C222_1$	752.8	12.0	0.16
205	AFO-N	$C222_1$	601.6	6.6	0.04
206	AFR-N	Pmmn	1343.6	12.9	0.20
207	AHT-N	$C222_1$	978.7	6.0	0.05
208	APC-N	Ia-3d	1414.5	7.3	0.08
209	APD-N	Pmna	656.9	5.8	0.02
210	ASV-N	Pbca	1137.0	9.7	0.11
211	ATN-N	I-4m2	686.7	8.1	0.05
212	ATS-N	Pbcn	1150.6	10.0	0.16
213	ATT-N	Pm2a	1424.6	7.7	0.10
214	ATV-N	C2mb	370.1	5.4	0.01
215	AWO-N	Cmca	1309.2	7.0	0.07
216	AWW-N	P4/nmm	1153.9	11.1	0.13
217	BCT-N	I-4m2	0.0	5.4	0.00
218	BEC-N	P42/m	1451.5	9.4	0.23
219	BIK-N	$C222_1$	796.3	6.0	0.04
220	BOF-N	Pnma	856.4	6.9	0.05
221	BOG-N	Imma	1284.2	13.1	0.21
222	BOZ-N	Pbcn			
223	CAS-N	$C222_1$	0.0	4.9	0.00
224	CDO-N	$C222_1$	995.8	7.3	0.06
225	CFI-N	Pmna	796.2	10.8	0.11
226	CGS-N	Pnma	1015.0	7.8	0.08
227	DFT-N	P42/m	1108.2	6.7	0.06
228	DON-N	Pbcn	763.6	11.5	0.16
229	EDI-N	P-4	1562.7	7.2	0.08
230	EEI-N	F222	0.0	9.8	0.00
231	ESV-N	Pnma	949.9	8.8	0.08
232	EUO-N	C2mb	945.7	9.8	0.11
233	EZT-N	Pnma	1058.5	9.5	0.13
234	FER-N	I222	1143.0	8.3	0.08
235	GIS-N	Ia-3d	0.0	7.7	0.00
236	GON-N	C222	699.5	8.7	0.06
237	GOO-N	$C222_1$	0.0	7.0	0.00
238	IFO-N	Pnnm	973.2	11.8	0.20

239	IHW-N	Pbca	800.9	8.4	0.07
240	IMF-N	Pbcn	1010.6	9.8	0.11
241	IRN-N	I222	1059.8	12.1	0.15
242	ITE-N	Cmcm	1417.1	11.9	0.18
243	ITH-N	Pnc2	1025.4	9.3	0.10
244	ITR-N	Pbcn	1146.9	8.6	0.11
245	IWR-N	C222	1438.2	11.7	0.22
246	IWV-N	F222	1378.9	13.5	0.28
247	IWW-N	Pba2	1294.5	10.5	0.16
248	JBW-N	$P2_122$	678.9	5.8	0.03
249	JOZ-N	$Pbc2_1$	834.6	5.9	0.03
250	JRY-N	$I2_12_12_1$	939.2	5.6	0.03
251	JSW-N	Pbca	0.0	7.5	0.00
252	LOV-N	P42/mmc	1940.0	7.1	0.10
253	LTJ-N	I4/mmm	0.0	3.8	0.00
254	MER-N	Pnma	1627.9	10.7	0.12
255	MFI-N	Pnn2	1016.1	9.3	0.09
256	MFS-N	Pbcn	903.2	8.8	0.08
257	MOR-N	Pmna	1016.5	9.0	0.09
258	MRE-N	$P2_12_12$	617.3	8.6	0.05
259	MTT-N	Pnn2	665.0	7.8	0.06
260	MVY-N	I-4	839.8	5.9	0.04
261	NAB-N	F222	2093.3	5.8	0.08
262	NES-N	F222	1089.2	10.1	0.14
263	NON-N	Immm	0.0	9.0	0.00
264	OBW-N	Pm2a	2920.3	13.8	0.45
265	OWE-N	Pbcn	1186.6	7.6	0.08
266	PHI-N	$Pca2_1$	964.9	6.6	0.04
267	PON-N	Pnnm	908.7	6.6	0.04
268	POS-N	Pbcn	1020.7	9.9	0.11
269	PUN-N	I222	2303.3	8.5	0.24
270	SAF-N	I-4m2	724.9	11.9	0.11
271	SAO-N	I4/m	1840.6	12.9	0.36
272	SAS-N	P4/nmm	1396.6	12.9	0.19
273	SAV-N	Immm	1718.3	13.2	0.20
274	SBE-N	$P2_122$	1757.6	19.7	0.38
275	SFG-N	$C222_1$	894.6	8.6	0.09
276	SFH-N	$C222_1$	952.2	12.7	0.18
277	SIV-N	C222	866.1	6.6	0.04
278	SZR-N	$C222_1$	1039.6	9.6	0.08
279	TER-N	$P2_122$	1179.2	8.8	0.10
280	THO-N	$C222_1$	1351.8	6.2	0.06
281	TON-N	Fmm2	652.9	6.7	0.04

282	UEI-N	Immm	0.0	7.0	0.00
283	UFI-N	Pm2a	1540.4	14.7	0.23
284	UOS-N	P-4	1010.5	7.4	0.06
285	VET-N	Pbcm	553.2	9.5	0.07
286	ZON-N	Ia-3d	1161.3	8.7	0.09
287	ANA-N	Pccn	0.0	5.4	0.00
288	AST-N	Fd-3m	0.0	5.9	0.00
289	FAU-N	Ia-3d	1664.1	17.1	0.42
290	GIE-N	Pa-3	1314.6	8.5	0.12
291	JSR-N	Pa-3	2864.7	12.8	0.54
292	JST-N	Im-3m	2915.6	9.1	0.34
293	KFI-N	P432	1478.2	16.3	0.22
294	LTA-N	Pm-3	1705.7	15.8	0.25
295	NPT-N	P222	2696.5	17.8	0.40
296	MEP-N	Pnnm	0.0	7.0	0.00
297	PCB-N	Im-3m	698.0	6.2	0.03
298	RHO-N	I-43m	1626.9	15.0	0.20
299	RWY-N	I432	3437.2	24.2	1.21
300	SOD-N	I432	2582.1	9.9	0.20

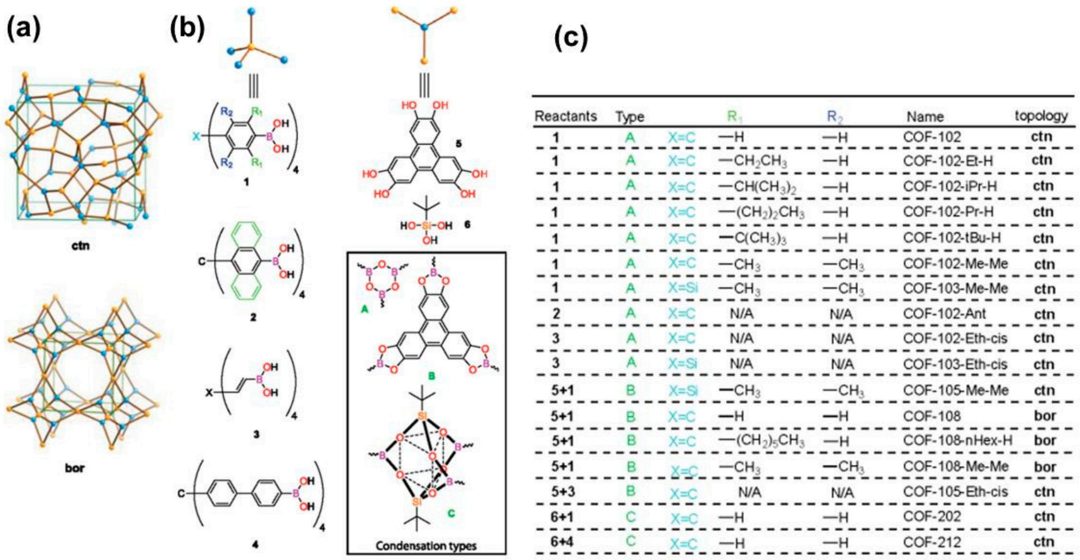

Figure S1. (a) ctn and bor topologies, (b) the building blocks for designing new COFs, (c) the reactions from various reactants to created new COFs.

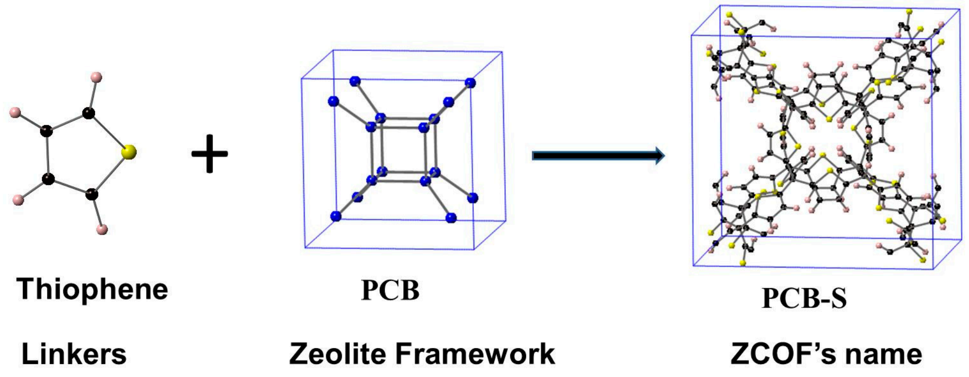

Figure S2. Design strategy of ZCOFs with thiophene linker. The spheres in black, pink, yellow denote C, H, and S atoms, respectively.

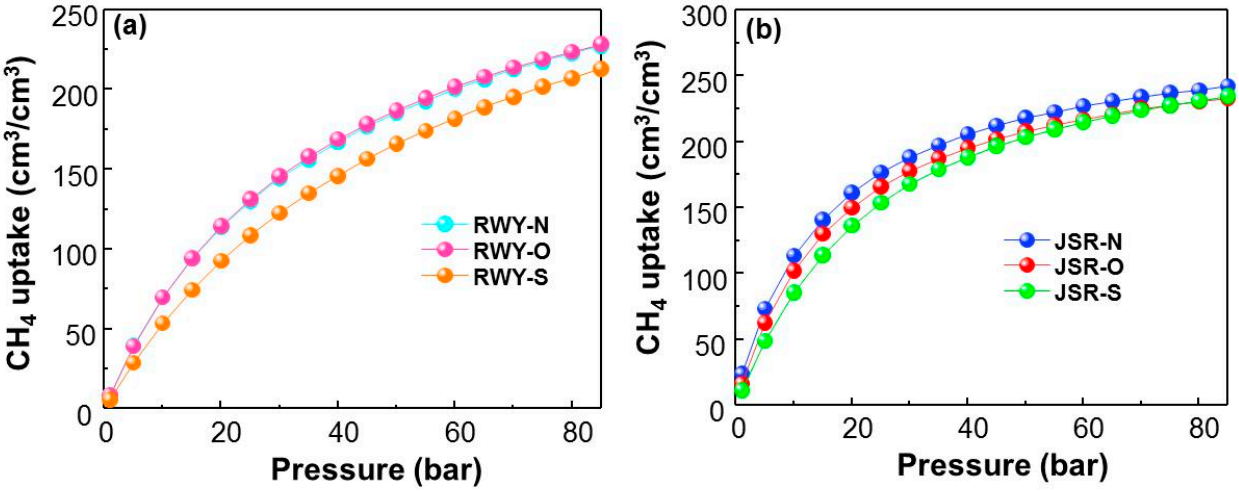

Figure S3. Isotherms of total volumetric CH_4 uptake at 298 K from 0 to 85 bar of (a) RWY-X, and (b) JRN-X (X = N, O, S).

Computer code

Input for calculation of frequency to determine thermal dynamic parameter

NPT-S:

```
COFNPT631G
EXTERNAL
FREQCALC
ANALYSIS
PRESSURE
1 0.101325 0.101325
TEMPERAT
1 298.15 298.15
ENDFREQ
END
1 2
```

```
0 0 3 1.0 1.0
     18.7311370          0.03349460
      2.8253937          0.23472695
      0.6401217          0.81375733
0 0 1 0.0 1.0
      0.1612778          1.0000000
6 3
0 0 6 2.0 1.0
   3047.5249000          0.0018347
    457.3695100          0.0140373
    103.9486900          0.0688426
     29.2101550          0.2321844
      9.2866630          0.4679413
      3.1639270          0.3623120
0 1 3 4.0 1.0
      7.8682724         -0.1193324          0.0689991
      1.8812885         -0.1608542          0.3164240
      0.5442493          1.1434564          0.7443083
0 1 1 0.0 1.0
      0.1687144          1.0000000          1.0000000
16 4
0 0 6 2.0 1.0
  21917.1000000          0.0186900
   3301.4900000          0.0142300
    754.1460000          0.0696960
    212.7110000          0.2384870
     67.9896000          0.4833070
     23.0515000          0.3380740
0 1 6 8.0 1.0
    423.7350000         -0.0023767          0.0040610
    100.7100000         -0.0316930          0.0306810
     32.1599000         -0.1133170          0.1304520
     11.8079000          0.0560900          0.3272050
      4.6311000          0.5922550          0.4528510
      1.8702500          0.4550060          0.2560420
0 1 3 6.0 1.0
      2.6158400         -0.2503740         -0.0145110
      0.9221670          0.0669570          0.3102630
      0.3412870          1.0545100          0.7544830
0 1 1 0.0 1.0
      0.1171670          1.0000000          1.0000000
99 0
```

```
END
DFT
EXCHANGE
PBE
CORRELAT
PBE
XLGRID
END
SCFDIR
BIPOSIZE
10000000
EXCHSIZE
20000000
SHRINK
2 2     S6
TOLINTEG
7 7 7 7 14
FMIXING
90
BROYDEN
0.05 50 5
TOLDEE
9
ENDSCF
```

JST-S:

```
COFJST631G
EXTERNAL
FREQCALC
ANALYSIS
PRESSURE
1 0.101325 0.101325
TEMPERAT
1 298.15 298.15
ENDFREQ
END
1 2
0 0 3 1.0 1.0
     18.7311370          0.03349460
      2.8253937          0.23472695
      0.6401217          0.81375733
0 0 1 0.0 1.0
```

```
        0.1612778                 1.0000000
6 3
0 0 6 2.0 1.0
    3047.5249000                0.0018347
     457.3695100                0.0140373
     103.9486900                0.0688426
      29.2101550                0.2321844
       9.2866630                0.4679413
       3.1639270                0.3623120
0 1 3 4.0 1.0
       7.8682724               -0.1193324               0.0689991
       1.8812885               -0.1608542               0.3164240
       0.5442493                1.1434564               0.7443083
0 1 1 0.0 1.0
       0.1687144                1.0000000               1.0000000
16 4
0 0 6 2.0 1.0
   21917.1000000                0.0186900
    3301.4900000                0.0142300
     754.1460000                0.0696960
     212.7110000                0.2384870
      67.9896000                0.4833070
      23.0515000                0.3380740
0 1 6 8.0 1.0
     423.7350000               -0.0023767               0.0040610
     100.7100000               -0.0316930               0.0306810
      32.1599000               -0.1133170               0.1304520
      11.8079000                0.0560900               0.3272050
       4.6311000                0.5922550               0.4528510
       1.8702500                0.4550060               0.2560420
0 1 3 6.0 1.0
       2.6158400               -0.2503740              -0.0145110
       0.9221670                0.0669570               0.3102630
       0.3412870                1.0545100               0.7544830
0 1 1 0.0 1.0
       0.1171670                1.0000000               1.0000000
99 0
END
DFT
EXCHANGE
PBE
CORRELAT
```

```
PBE
XLGRID
END
SCFDIR
BIPOSIZE
10000000
EXCHSIZE
20000000
SHRINK
2 2     S6
TOLINTEG
7 7 7 7 14
FMIXING
90
BROYDEN
0.05 50 5
TOLDEE
9
ENDSCF
```

Input for GCMC

```
------ General Information -----------------------------------------
CH4 molecule in JSRN
15000000                # No. of iterations
5000000                 # No. of steps between writes to output/log file
1000000                 # No. of steps between writes to crash file
50000                   # No. of steps between writes to config. file
1                       # Start numbering simulations from .
635535                  # Iseed
4                       # specifies contents of config file,
JSRN.CH4.res            # Restart File to write to
JSRN.CH4.con            # Configuration File
------ Atomic Types ------------------------------------------------
4            # number of atomic types

CH4                     # atom type
CH4.atm                 # basic atom info file

H                       # atom type
H.atm                   # basic atom info file

C                       # atom type
C.atm                   # basic atom info file
```

```
N                    # atom type
N.atm                # basic atom info file
------ Molecule Types -----------------------------------------------
2                          # number of sorbate types

CH4                    # sorbate
CH4.mol                # sorbate coordinates file

JSRN                       # sorbate
JSRN.mol                   # sorbate coordinates file
------ Simulation Cell Information -----------------------------------
JSRN                       # Fundamental cell file
2, 2, 2                  # No. of unit cells in x, y, z direction
1, 1, 1                  # (1 = Periodic) in x, y, z
------ Forcefield Information -----------------------------------------
BASIC
SPC
atom_atom_file       # atom-atom interaction file
sorb_sorb_file       # sorbate-sorbate interaction file
intramolecular_file   # intramolecular interaction file/specification
------ Ideal Parameters ----------------------------------------------
Ideal                      # Equation of State
1                          # no. of sorbates
CH4                    # Sorbate Name
------ GCMC Information ----------------------------------------------
1                      # No. of iterations
298.0 # temperature
Ideal Parameters     # Tag for the equation of state (NULL = Ideal Gas)
19                         # No. of simulation points
5000                       # Block size for statistics
1                        # no. of sorbates
               ------------------------
CH4                  # Sorbate Name
fugacity.dat   #   pressure
Null                     # sitemap filename (Null = no sitemap)
3                        # no of gcmc movetypes
1.0, 1.0, 1.0      # move type weights
BINSERT                            # type of move.1
JSRN.CH4.pmap
298.0                              # Bias temperature for the bmap
BDELETE
```

RTANSLATE # type of move.4
0.2, 0 # Delta Translate, adjust delta option (0=NO, 1=YES)
------ Configuration Initialization -------------------------------------
CH4 # Sorbate_Type
GCMC NULL
JSRN # Sorbate_Type
FIXED NULL
-------- Main Datafile Information --------
Energy, position, pair_energy # contents of datafile
